# Normal Adrenal and Thyroid Function in Patients Who Survive COVID-19 Infection

**DOI:** 10.1210/clinem/dgab349

**Published:** 2021-05-19

**Authors:** Sophie A Clarke, Maria Phylactou, Bijal Patel, Edouard G Mills, Beatrice Muzi, Chioma Izzi-Engbeaya, Sirazum Choudhury, Bernard Khoo, Karim Meeran, Alexander N Comninos, Ali Abbara, Tricia Tan, Waljit S Dhillo

**Affiliations:** 1 Division of Diabetes, Endocrinology and Metabolism, Department of Metabolism, Digestion and Reproduction, Imperial College London, London, W12 0NN, UK; 2 Department of Endocrinology, Imperial College Healthcare NHS Trust, London, W6 8RF, UK; 3 Department of Clinical Biochemistry, Imperial College Healthcare NHS Trust, London, W6 8RF, UK; 4 Department of Endocrinology, Division of Medicine, Faculty of Medical Sciences, Royal Free Campus, University College London, London, NW3 2QG, UK

**Keywords:** COVID-19, SARS-CoV-2, adrenal insufficiency, adrenal function, thyroid function, thyroid gland

## Abstract

**Context:**

The COVID-19 pandemic continues to exert an immense burden on global health services. Moreover, up to 63% of patients experience persistent symptoms, including fatigue, after acute illness. Endocrine systems are vulnerable to the effects of COVID-19 as many glands express the ACE2 receptor, used by the SARS-CoV-2 virion for cellular access. However, the effects of COVID-19 on adrenal and thyroid gland function after acute COVID-19 remain unknown.

**Objective:**

Our objectives were to evaluate adrenal and thyroid gland function in COVID-19 survivors.

**Methods:**

A prospective, observational study was undertaken at the Clinical Research Facility, Imperial College NHS Healthcare Trust, including 70 patients ≥18 years of age, at least 3 months after diagnosis of COVID-19. Participants attended a research study visit (8:00-9:30 am), during which a short Synacthen test (250 µg IV bolus) and thyroid function assessments were performed.

**Results:**

All patients had a peak cortisol ≥450 nmol/L after Synacthen, consistent with adequate adrenal reserve. Basal and peak serum cortisol did not differ according to disease severity or history of dexamethasone treatment during COVID-19. There was no difference in baseline or peak cortisol after Synacthen or in thyroid function tests, or thyroid status, in patients with fatigue (n = 44) compared to those without (n = 26).

**Conclusion:**

Adrenal and thyroid function ≥3 months after presentation with COVID-19 was preserved. While a significant proportion of patients experienced persistent fatigue, their symptoms were not accounted for by alterations in adrenal or thyroid function. These findings have important implications for the clinical care of patients after COVID-19.

As the COVID-19 pandemic continues (1), questions regarding its consequences on the endocrine system remain (2). The route by which severe acute respiratory syndrome coronavirus 2 (SARS-CoV-2), the virus responsible for COVID-19, accesses cells (via angiotensin-converting enzyme 2 [ACE2] receptors) renders the endocrine system potentially susceptible to damage by COVID-19, as ACE2 receptors are expressed in the adrenal gland, thyroid gland, and testes (3, 4). Some evidence of the adverse effects on endocrine function by coronavirus infections comes from SARS, the precursor to COVID-19, caused by another coronavirus, SARS-CoV. Postmortem examination of patients with SARS revealed SARS-CoV RNA in the pituitary gland (5), in addition to destruction of thyroid follicular and parafollicular cells (6), and a longitudinal study of survivors of SARS showed that up to 39% had hypocortisolism (7). In patients with COVID-19, microscopic adrenal lesions in postmortem specimens (8, 9), adrenal hemorrhage (10, 11), and infarction (12) have been reported. There is also the potential for iatrogenic impairment of adrenal function, as therapeutic glucocorticoid treatment has been routinely used since July 2020 to treat patients with COVID-19 requiring oxygen therapy or ventilatory support (13), raising the possibility of tertiary adrenal insufficiency (14).

It is increasingly evident that the health impact of COVID-19 extends beyond the initial infection, with up to 63% of patients reporting ongoing symptoms, including fatigue (15, 16). The pathophysiology of this “post-COVID syndrome” is currently poorly understood. Adrenal and thyroid gland dysfunction are known to be associated with fatigue (17) and may additionally present with other components of the post-COVID syndrome, including autonomic dysfunction and cognitive impairment (18, 19). As these endocrinopathies are eminently treatable, it is imperative to identify any contribution they may have to the persistent symptoms experienced by patients after COVID-19 infection.

Therefore, our objectives in this study were to evaluate adrenal and thyroid function in survivors of COVID-19, and to investigate the relationship between them and the symptom of persistent fatigue experienced post-COVID. We addressed this by investigating adrenal and thyroid function prospectively in patients ≥3 months after initial diagnosis of COVID-19.

## Methods

### Ethical Approval

This study was performed in accordance with the Declaration of Helsinki. All participants provided written informed consent prior to inclusion in the study. Ethical approval for this study was granted by the London Bridge Research Ethics Committee (REC ref 20/HRA/4110). This study was registered with ISRCTN (ISRCTN15615697).

### Study Design and Participants

In this prospective study, participants were recruited from a cohort of patients who attended Imperial College London NHS Healthcare Trust with a clinical suspicion of COVID-19 between March and November 2020. Additional participants were also recruited via advertisements placed in social media for patients who had tested positive for COVID-19 ≥3 months prior to their inclusion in the study. All patients aged ≥18 years with a diagnosis of COVID-19 confirmed using either real-time RT-PCR testing of a nasopharyngeal swab, confirmatory imaging (chest radiograph or computed tomography scan), or a positive serum SARS-CoV-2 IgG antibody test taken after symptom onset were eligible for inclusion. Patients who were prescribed steroids (oral, inhaled, topical or intra-articular) following recovery from COVID-19, and those taking other medications known to affect cortisol-binding globulin (including oral estrogens) were excluded from the study. Similarly, those patients with underlying health conditions or states known to influence cortisol-binding globulin (including pregnancy, end-stage renal failure, or underlying malignancy) were also excluded.

### Study Protocol

Participants were invited to attend for their study visit at least 3 months following their initial presentation with COVID-19. Participants were nonfasted and study visits commenced between 8:00 and 9:30 am. Study visits comprised a clinical assessment, including medical history and physical examination, and a urinary pregnancy test in women of reproductive age. During the clinical assessment, patients were asked regarding the presence of persistent symptoms following COVID-19 (including cough, shortness of breath, chest pain, low-grade fever, fatigue, headache, difficulty concentrating, muscle aches, loss of appetite, nausea, diarrhea, rash, or change in mood). In the absence of a validated measure of fatigue in patients post-COVID-19 at the time of study design, rates of fatigue were initially quantified using the “yes/no” response to the direct question of experience of fatigue, consistent with other studies investigating COVID-19 (20, 21) and other disease paradigms (22, 23). All participants were subsequently asked to rate the frequency of fatigue experienced following acute COVID-19 on a scale of 0 to 4 (0 = none of the time, 1 = a little of the time, 2 = about half of the time, 3 = most of the time, 4 = all of the time) and severity of fatigue on a scale of 0 to 4 (0 = symptom not present, 1 = mild, 2 = moderate, 3 = severe, 4 = very severe). The severity of COVID-19 was classified by applying World Health Organization (WHO) classification criteria (24) to parameters from participants’ presentation with COVID-19 by 2 independent clinicians (S.A.C. and M.P.) who were not involved in the clinical care of participants during their presentation with COVID-19. Where discrepancies occurred, the least severe classification was selected. Additionally, severity was also assessed according to the level of care provided. A cannula was inserted and baseline samples taken for serum cortisol, thyroid-stimulating hormone (TSH), free thyroxine (fT4) and free triiodothyronine (fT3), and plasma adrenocorticotropic hormone (ACTH). Additionally, samples were also taken for SARS-CoV-2 antibody testing. Following this, 250 µg tetracosactide (Synacthen) was injected intravenously. Samples for cortisol were taken at 30 and 60 minutes after injection.

### Interpretation of Short Synacthen Test

Participants were considered to have an adequate response to Synacthen if they reached a peak cortisol value of ≥ 450 nmol/L and increment by ≥150 nmol/L from baseline, either at 30 or 60 minutes after Synacthen (18, 25). Although a lower reference limit of peak cortisol ≥ 430 nmol following 250 µg Synacthen has been proposed for the Abbott cortisol assay (25), our local policy is to use a more conservative cortisol cutoff of 450nmol/L to denote adequate response.

### Assay Methodology

Serum cortisol, TSH, fT4, and fT3 were measured using Abbott Alinity ci-series analyzer using chemiluminescent microparticle immunoassays. The lower limits of detection were as follows: cortisol, 19.3 nmol/L; TSH, 0.01 mU/L; fT4, 5.4 pmol/L; and fT3, 1.46 pmol/L. Interassay coefficients of variation were: cortisol, ≤ 5.1%; TSH, ≤ 2.1%; fT4, ≤ 3%; and fT3, ≤ 4.8%. Intra-assay coefficients of variation were: cortisol, ≤ 4.3%; TSH, ≤ 2.1%; fT4, ≤ 3%; and fT3, ≤ 3.8%. Serum ACTH was measured using Siemens Immulite. The inter- and intra-assay coefficient of variation was ≤10%. Reference ranges were as follows: TSH, 0.3 to 4.2 mU/L; fT4, 9 to 23 pmol/L; fT3, 2.5 to 5.7 pmol/L. Serum antibodies to SARS-CoV-2 N protein (IgG) were measured using Abbott Architect assay. For those with indeterminate result, additional testing for antibodies to the receptor binding domain of SARS-CoV-2 spike protein (IgG) was performed using Imperial Hybrid DABA (26).

### Outcomes

The primary outcome of this observational study was the number of patients with an insufficient response to intravenous (IV) Synacthen 250 µg ≥ 3 months after presentation with COVID-19 (25). The secondary outcomes were the TSH, fT4, and fT3 levels measured ≥ 3 months after presentation with COVID-19. Post hoc subgroup comparisons were made for differences in those reporting persistent fatigue on direct questioning (vs those who did not) and those who received dexamethasone as part of their treatment for COVID-19 (vs those who did not).

### Statistical Analysis

Data were analyzed using GraphPad Prism version 9.0. Data distribution was assessed using D’Agostino and Pearson, Kolmogorov-Smirnov tests, and Q-Q plots. Parametric data were presented as mean ± SD, whereas nonparametric data were presented as median with interquartile range (IQR). Continuous data that were parametrically distributed were compared using a Student *t* test for 2 groups, or one-way analysis of variance (ANOVA) for 3 or more groups. Continuous data that were nonparametrically distributed were compared using Mann-Whitney U tests for 2 groups, or Kruskal-Wallis test with post hoc Dunn’s test for 3 or more groups. Categorical data were compared using chi-squared test. Relationship between 2 continuous variables was determined using Pearson’s correlation, for parametrically distributed data, and Spearman’s rank correlation for nonparametrically distributed data.

## Results

### Baseline Characteristics

**Figure 1.**
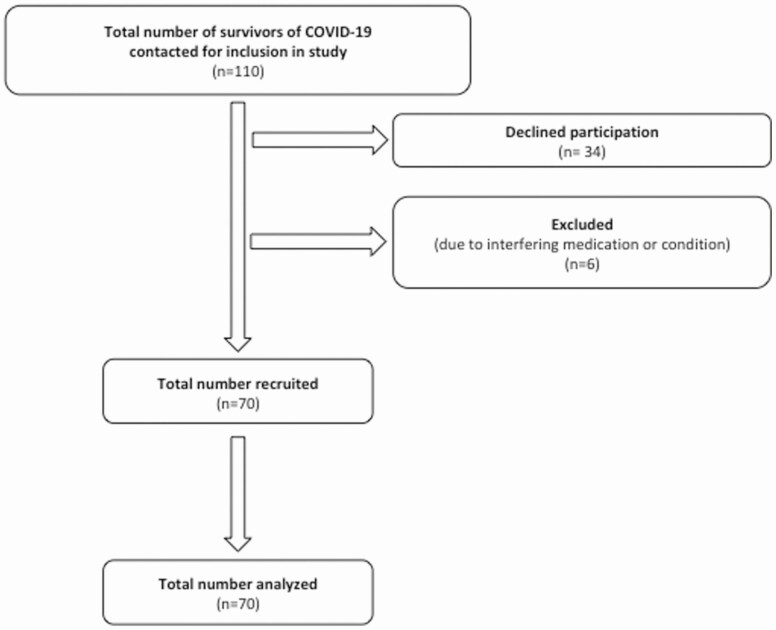
STROBE diagram showing patients meeting inclusion criteria (n = 70).

In total, 110 survivors of COVID-19 presenting from March to November 2020 were considered for inclusion to this study, of whom 40 were excluded either due to taking medications or having conditions known to interfere with the short Synacthen test (n = 6) or because they declined to take part (n = 34) (Fig. 1), resulting in 70 participants being enrolled in the study. The mean age (SD) was 55.9 (±13.0) years, and 67.1% of the study population were male (Table 1). Of this cohort, 77.1% of patients had been hospitalized; 22.0% of those hospitalized had also required either noninvasive ventilation or intensive care treatment (Table 1), and 31.4% received dexamethasone as part of their acute treatment for COVID-19. The median (IQR) duration of admission was 5 (1, 8) days. Patients attended for their research study visit appointment at a median (IQR) of 210 (112, 261) days following presentation (Table 1). Those who received dexamethasone attended their clinical research appointment sooner than those who did not (median [IQR] days following presentation: no dexamethasone 242 [209.5, 287.0], dexamethasone 95.5 [88.8, 113.3], *P* < 0.001) reflecting the fact that dexamethasone treatment was mandated in UK protocols for treatment of COVID-19 from July 2020 onward. On direct questioning, 62.9% (n = 44) of patients reported fatigue.

**Table 1:** Baseline characteristics of patients attending ≥ 3 months following presentation.

Participant characteristics	Total Cohort (n=70)	No Dexamethasone (n=48)	Dexamethasone (n=22)	*P* value	No fatigue (n=26)	Fatigue (n=44)	*P* value
**Age (years)**	55.9 (13.0)	55.6 (13.3)	56.6 (12.6)	.77	61.1 (11.6)	52.8 (12.9)	.009
***Sex:***							
**Male**	47/70 (67.1%)	33/48 (68.8%)	14/22 (63.6%)	.79	23/26 (88.5%)	24/44 (54.5%)	.004
**Female**	23/70 (32.9%)	15/48 (31.3%)	8/22 (36.4%)		3/26 (11.5%)	20/44 (45.5%)	
***Ethnicity:***							
**Asian**	17/70 (24.3%)	7/48 (14.6%)	10/22 (45.5%)	.006	8/26 (30.8%)	9/44 (20.5%)	.82
**Black**	7/70 (10.0%)	4/48 (8.3%)	3/22 (13.6%)		3/26 (11.5%)	4/44 (9.1%)	
**Mixed**	2/70 (2.9%)	1/48 (2.1%)	1/22 (4.6%)		1/26 (3.9%)	1/44 (2.3%)	
**Other – not stated**	17/70 (24.3%)	17/48 (35.4%)	0 (0.0%)		5/26 (19.2%)	12/44 (27.3%)	
**White**	27/70 (38.6%)	19/48 (39.6%)	8/22 (36.4%)		9/26 (34.6%)	18/44 (40.9%)	
***Comorbidities:***							
**Hypertension**	24/70 (34.3%)	13/48 (27.1%)	11/22 (50.0%)	.05	6/26 (23.1%)	18/44 (40.9%)	.20
**Cardiovascular disease**	4/70 (5.7%)	4/48 (8.3%)	0/22 (0.0%)		3/26 (11.5%)	1/44 (2.3%)	
**Diabetes**	20/70 (28.6%)	11/48 (22.9%)	9/22 (40.9%)		7/26 (26.9%)	13/44 (29.5%)	
** Type 1**	0/30 (0.0%)	0/11 (0.0%)	0/9 (0.0%)		0/7 (0.0%)	0/13 (0.0%)	
** Type 2**	18/20 (90.0%)	10/11 (90.9%)	8/9 (88.9%)		6/7 (85.7%)	13/13 (100.0%)	
** Unspecified**	2/20 (10.0%)	1/11 (90.1%)	1/9 (11.1%)		1/7 (14.3%)	0/13 (0.0%)	
**Obesity (BMI >30kg/m** ^**2**^)							
** Yes**	15/70 (21.4%)	6/48 (12.5%)	9/22 (40.9%)		4/26 (15.4%)	11/44 (25.0%)	
** No**	30/70 (42.9%)	20/48 (41.7%)	10/22 (45.5%)		17/26 (65.4%)	13/44 (29.5%)	
** Unknown**	25/70 (35.7%)	22/48 (45.8%)	3/22 (13.6%)		5/26 (19.2%)	20/44 (45.5%)	
***Smoking status:***							
**Current smoker**	2/70 (2.9%)	1/48 (2.1%)	1/22 (4.5%)	.23	2/26 (7.7%)	0/44 (0.0%)	.16
**Ex-smoker**	6/70 (8.6%)	6/48 (12.5%)	0/22 (0.0%)		3/26 (11.5%)	3/44 (6.8%)	
**Never-smoked**	60/70 (85.7%)	39/48 (81.3%)	21/22 (95.5%)		21/26 (80.8%)	39/44 (88.6%)	
**Unknown**	2/70 (2.9%)	2/48 (4.2%)	0/22 (0.0%)		0/26 (0.0%)	2/44 (4.6%)	
***Disease outcome:***							
**Hospitalized**	54/70 (77.1%)	32/48 (66.7%)	22/22 (100.0%)	NA	17/26 (65.4%)	37/44 (84.1%)	.07
** NIV**	5/54 (9.3%)	1/32 (3.1%)	4/22 (18.2%)		0/26 (0.0%)	5/37 (13.5%)	
** ITU admission**	7/54 (13.00%)	3/32 (9.4%)	4/22 (18.2%)		3/26 (11.5%)	4/37 (10.8%)	
**Non-hospitalized**	16/70 (22.9%)	16/48 (33.3%)	0/22 (0.0%)		9/26 (34.6%)	7/44 (15.9%)	
***Disease Severity:***							
**Mild**	12/70 (17.1%)	12/48 (25.0%)	0/22 (0.0%)	.04	8/26 (30.8%)	4/44 (9.1%)	.03
**Moderate**	30/70 (42.9%)	19/48 (39.6%)	11/22 (50.0%)		6/26 (23.1%)	24/44 (54.5%)	
**Severe**	21/70 (30.0%)	14/48 (29.2%)	7/22 (31.8%)		9/26 (34.6%)	12/44 (27.3%)	
**Critical**	7/70 (10.0%)	3/48 (6.3%)	4/22 (18.2%)		3/26 (11.5%)	4/44 (9.1%)	
**Dexamethasone treatment**	22/70 (31.4%)	NA	22/22 (100%)	NA	0/26 (0.0%)	22/44 (50.0%)	NA
**Cumulative dose dexamethasone treatment (mg)**	38.73 (18.49)	NA	38.73 (18.49)	NA	NA	38.73 (18.49)	NA
***Additional treatments:***							
** Remdesivir**	15/70 (21.4%)	1/48 (2.1%)	14/22 (63.6%)	.05	8/26 (30.8%)	7/44 (15.9%)	.26
** Tocilizumab**	1/70 (1.4%)	1/48 (2.1%)	0/22 (0.0%)		1/26 (3.9%)	0/44 (0.0%)	
** Conv. Plasma**	2/70 (2.9%)	0/48 (0.0%)	2/22 (9.1%)		1/26 (3.9%)	1/44 (2.3%)	
** Other**	3/70 (4.3%)	1/48 (2.1%)	2/22 (9.1%)		0/26 (0.0%)	3/44 (6.8%)	
**Duration of admission (days)**	5.0 (1.0, 8.0)	4.0 (0.0, 8.0)	6.0 (4.0, 9.8)	<.001	6.5 (3.0, 1.3)	4.0 (0.0, 7.8)	.03
**Time since presentation (days)**	210.0 (112.0, 261.0)	242.0 (209.5, 287.0)	95.5 (88.8, 113.3)	<.001	215.00 (121.0, 252.3)	209.0 (102.0, 281.0)	.83

Data are means (SD) for continuous variables parametrically distributed and medians (lower quartile, upper quartile) for continuous non-parametrically distributed variables. For categorical data, numbers of patients (percentages) are presented. Continuous data that are parametrically distributed were compared using t-tests, continuous data not parametrically distributed were compared using Mann-Whitney U test. Groups of continuous data parametrically distributed were compared using ANOVA test. Groups of continuous data non-parametrically distributed were compared using Kruskal-Wallis test. Categorical data were compared using Chi-Squared test. NIV: non-invasive ventilation; ITU: intensive therapy unit, Conv Plasma: convalescent plasma; Other: additional treatments include Anakinra (interleukin-1 receptor antagonist), Kaletra (Lopinavir/Ritonavir), Namilumab; NA: not applicable.

### Adrenal Function is Preserved After COVID-19

**Figure 2.**
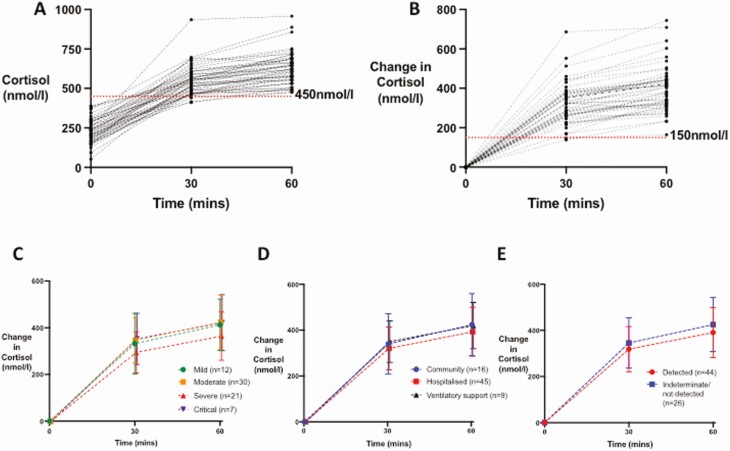
Serum cortisol response to Synacthen 250 µg in patients 3 months after COVID-19 (n = 70). (A) Individual responses of serum cortisol at 30 and 60 minutes after an IV bolus of 250 µg of Synacthen in patients ≥3 months after presentation with COVID-19 are presented. (B) Individual responses in change in serum cortisol from baseline at 30 and 60 minutes after an IV bolus of 250 µg of Synacthen in patients assessed ≥3 months after presentation with COVID-19 are presented. (C) Mean and SD change in cortisol from baseline (nmol/L) after an IV bolus of 250 µg of Synacthen in patients recovering from COVID-19 by WHO Disease Severity (mild, n = 12 represented by green symbols and line; moderate, n = 30, represented by orange symbols and lines; severe, n = 21, represented by red symbols and line; critical, n = 7, represented by purple symbols and line). (D) Mean (error bars show SD) change in cortisol from baseline (nmol/L) after Synacthen 250 µg in patients recovering from COVID-19 by level of care required: community care (n = 16), represented by blue symbols and line; hospitalized and not requiring ventilatory support (n = 45), represented by red symbols and lines; ventilatory support required (n = 9), represented by black symbols and lines. (E) Mean and SD change in cortisol from baseline (nmol/L) after Synacthen 250 µg in patients recovering from COVID-19 by antibody status as determined by Abbott Architect IgG to SARS-CoV-2 (detected, n = 44, represented by blue symbols and lines; not detected/indeterminate, n = 26, represented by red symbols and lines).

At follow-up ≥ 3 months after initial presentation with COVID-19, mean (± SD) baseline cortisol was 233.1 (± 77.5) nmol/L with a median (IQR) basal ACTH of 14.85 (8.34, 22.10) ng/L. All patients achieved a peak cortisol of ≥450 nmol/L post-Synacthen (Fig. 2), with a mean cortisol at 30 minutes post-Synacthen of 562.1 (87.6) nmol/L and mean cortisol at 60 minutes post-Synacthen of 636.6 (97.6) nmol/L (Fig. 2A). Baseline and peak cortisol (nmol/L) were not related to time from admission (baseline cortisol vs time from admission Pearson ρ −0.09, *P = *0.45; peak cortisol vs time from admission Pearson ρ −0.09, *P* = 0.46; Supplemental Fig. 1A and 1B (27)). Of those with available data (n = 24) (Admission Cohort, see Table 2), there was no relationship between cortisol taken within the first 48 hours of acute admission with COVID-19, and either baseline cortisol or peak cortisol following Synacthen at follow-up endocrine testing (baseline cortisol vs admission cortisol, Pearson ρ 0.21, *P = *0.34; peak cortisol vs admission cortisol, Pearson ρ 0.24, *P = *0.25; Supplemental Fig. 1C and 1D (27)). Subgroup analysis did not reveal any difference in response to Synacthen with disease severity, as determined by WHO classification (Table 2, Fig. 2C), level of care received (Fig. 2D), or antibody status (Fig. 2E).

**Table 2.** Hormonal data of patients attending ≥3 months following presentation with COVID-19

	Total cohort (n = 70)	No Dex (n = 48)	Dex (n = 22)	*P* value	No Fatigue (n = 26)	Fatigue (n = 44)	*P* valve	Mild (n = 12)	Moderate (n = 30)	Severe (n = 21)	Critical (n = 7)	*P* value
Baseline cortisol (nmol/L)	233.1 (77.5)	229.3 (75.7)	241.6 (82.5)	0.54	243.8 (84.9)	227.2 (73.5)	0.40	216.9 (64.2)	224.8 (85.0)	268.2 (72.4)	191.3 (44.7)	0.06
30-min cortisol (nmol/L)	562.1 (87.6)	549.3 (93.1)	591.2 (66.6)	0.81	551.4 (63.5)	568.1 (98.9)	0.45	549.1 (135.2)	571.5 (84.8)	562.5 (59.1)	543.9 (84.5)	0.83
60-min cortisol (nmol/L)	636.6 (97.6)	623.3 (105.5)	665.8 (71.4)	0.78	623.0 (89.9)	644.2 (101.8)	0.34	629.3 (127.3)	648.0 (101.0)	632.5 (79.1)	613.0 (90.5)	0.83
30-min change in cortisol (nmol/L)	328.8 (102.4)	320.0 (103.9)	348.7 (98.6)	0.29	307.7 (87.7)	340.7 (109.1)	0.20	332.2 (130.0)	346.5 (96.9)	294.3 (87.9)	352.6 (109.8)	0.31
60-min change in cortisol (nmol/L)	403.5 (112.30	394.0 (119.3)	424.2 (94.6)	0.30	379.2 (112.1)	417.0 (111.4)	0.18	412.4 (110.4)	423.1 (116.0)	364.3 (103.2)	421.7 (119.8)	0.30
ACTH (ng/L)	14.85 (8.34, 22.10)	16.05 (9.05, 22.13)	14.05 (7.53, 21.75)	0.54	19.15 (14.03, 22.80)	12.35 (7.55, 21.55)	0.06	9.35 (6.38, 12.43)	17.75 (7.48, 22.25)	21.20 (11.75, 27.10)	15.00 (8.30, 20.40)	0.01
TSH (mU/L)	1.32 (0.97, 2.09)	1.42 (1.02, 2.13)	1.24 (0.80, 1.82)	>0.99	1.33 (1.03, 2.09)	1.36 (0.96, 2.10)	0.95	1.60 (0.90, 2.53)	1.29 (1.06, 1.79)	1.32 (0.84, 2.07)	1.24 (1.03, 3.03)	0.84
fT4 (pmol/L)	12.30 (11.65, 13.08)	12.20 (11.75, 12.73)	12.40 (11.55, 13.60)	1.00	12.30 (11.68, 13.30)	12.25 (11.65, 13.03)	0.65	12.05 (11.60, 12.70)	12.30 (11.80, 12.90)	12.40 (11.83, 13.60)	11.20 (10.60, 12.30)	0.15
fT3 (pmol/L)	4.40 (4.08, 4.80)	4.40 (4.00, 4.80)	4.30 (4.18, 4.63)	1.00	4.40 (4.20, 4.80)	4.20 (3.90, 4.70)	0.14	4.15 (3.93, 4.48)	4.30 (3.90, 4.83)	4.40 (4.15, 4.80)	4.60 (4.20, 4.70)	0.34
	**Admission cohort (n = 24)**	**No Dex (n = 24)**	**Dex (n = 0)**	***P* value**	**No Fatigue (n = 11)**	**Fatigue (n = 13)**	***P* value**	**Mild (n = 0)**	**Moderate (n = 14)**	**Severe (n = 9)**	**Critical (n = 1)**	***P* value**
Admission cortisol (nmol/L)	566 (428.5, 749.3)	566 (428.5, 749.3)	NA	NA	650.0 (505.0, 1194.0)	489.0 (407.5, 657.5)	0.04	NA	472.5 (393.8, 555.5)	760.0 (664.0, 1205)	1158	NA

Data are means (SD) for continuous variables parametrically distributed and medians (lower quartile, upper quartile) for continuous variables nonparametrically distributed. For categorical data, numbers of patients (percentages) are presented. Continuous data that are parametrically distributed were compared using *t* tests, continuous data not parametrically distributed were compared using Mann-Whitney U test. Groups of data parametrically distributed were compared using ANOVA test. Groups of data nonparametrically distributed were compared using Kruskal-Wallis test.

Abbreviations: ACTH, adrenocorticotropic hormone; Dex, dexamethasone treatment; fT3, free triiodothyronine; fT4, free thyroxine; NA, not applicable; no Dex, no dexamethasone treatment; TSH, thyroid-stimulating hormone.

**Figure 3.**
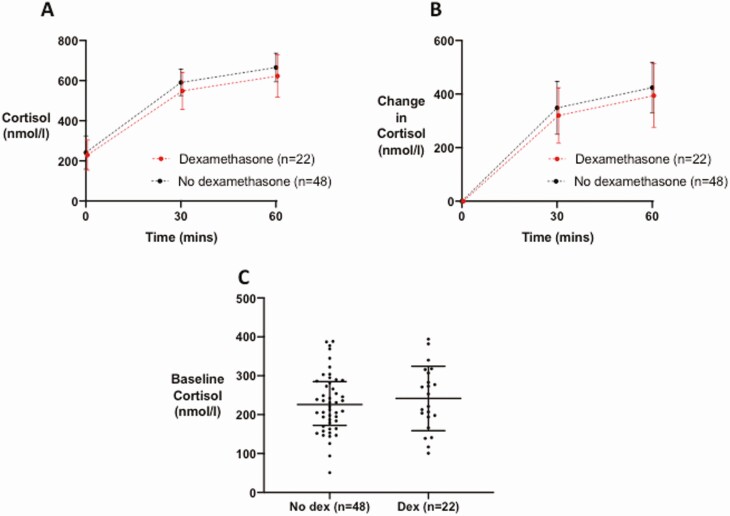
Serum cortisol response to 250 µg Synacthen in patients who received dexamethasone as part of their treatment for COVID-19 (n = 22, represented by red symbols and lines) vs those who did not (n = 48, represented by black symbols and lines). (A) Data presented are mean (error bars show SD) cortisol (nmol/L) at 30 and 60 minutes following Synacthen 250 µg. (B) Data presented are mean (error bars show SD) change in serum cortisol from baseline (nmol/L) at 30 and 60 minutes following Synacthen 250 µg. (C) Graph of baseline serum cortisol (nmol/L).

Treatment protocols mandating dexamethasone for the treatment of COVID-19 requiring supplemental oxygen or assisted ventilation were introduced in the UK in July 2020, following initial study design. A post hoc analysis of cortisol response in patients who received dexamethasone (n = 22), did not reveal any difference in cortisol response at either 30 or 60 minutes post-Synacthen compared with those who did not (Table 2, Fig. 3A and 3B). Similarly, basal cortisol was not different between the 2 groups (Fig. 3C).

### Adrenal Function Is Preserved in Patients With Persistent Fatigue Following COVID-19

**Figure 4.**
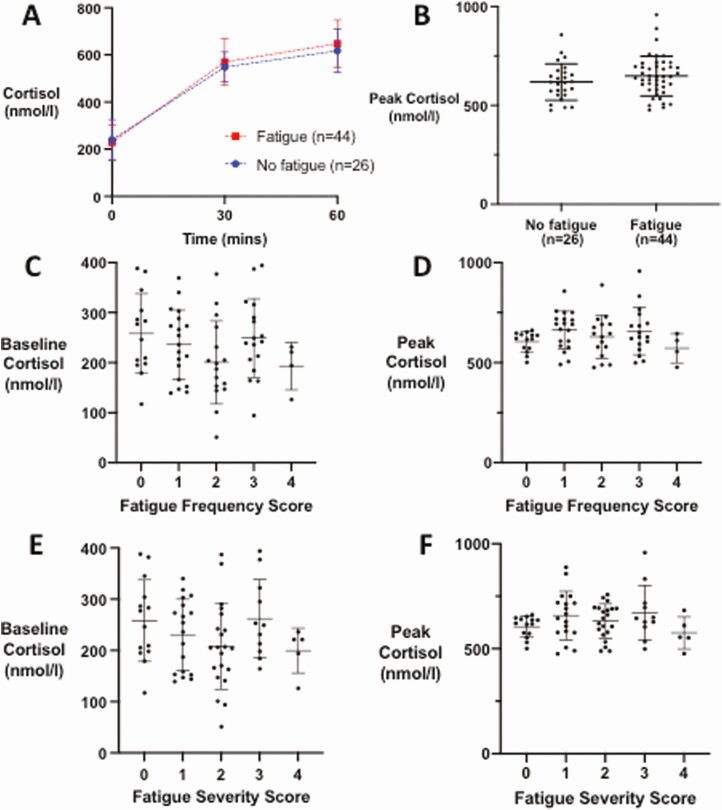
Serum cortisol response to 250 µg Synacthen in patients 3 months after COVID-19 with persistent fatigue (n = 44) compared with those without (n = 26). (A) Mean (error bars show SD) for serum cortisol (nmol/L) at 30 and 60 minutes following Synacthen, comparing patients with persistent fatigue (n = 44, red lines and symbols) vs those with no persistent fatigue (n = 26, blue lines and symbols). (B) Graph of peak serum cortisol in patients with persistent fatigue and those without persistent fatigue. Individual data points plotted with horizontal line representing mean, whiskers represent SD. (C) Graph of baseline cortisol in groups classified by tiredness frequency graded 0 (not present), 1 (present a little of the time), 2 (present about half of the time), 3 (present most of the time), 4 (present all of the time). (D) Graph of peak cortisol in groups classified by tiredness frequency graded 0 (not present), 1 (present a little of the time), 2 (present about half of the time), 3 (present most of the time), 4 (present all of the time). (E) Graph of baseline cortisol in groups classified by tiredness severity graded 0 (not present), 1 (mild), 2 (moderate), 3 (severe), 4 (very severe). (F) Graph of peak cortisol (nmol/L) in groups classified by tiredness severity score graded 0 (not present), 1 (mild), 2 (moderate), 3 (severe), 4 (very severe).

In our cohort, post hoc analysis revealed that 62.9% (n = 44) of patients reported persistent fatigue on direct questioning ≥ 3 months after presentation with COVID-19 (Fatigue cohort), with median (IQR) time since presentation of 209 (102, 281) days (Table 1). In the Fatigue cohort there was a slight predominance of male patients reflecting the composition of the cohort as a whole (male gender in total cohort 67.1% vs 54.5% in the Fatigue cohort), but in the No fatigue cohort, there was a more marked predominance of male patients (No fatigue 88.5% vs Fatigue 54.5%, *P =* 0.004). The patients in the Fatigue cohort were younger (mean ± SD age 52.8 ± 12.9 years, vs 61.1 ± 11.6 years for the No fatigue cohort, *P = *0.009) (Table 1). In the fatigue cohort, 27.3% had had severe COVID-19 illness, compared with 34.6% of those in the No fatigue cohort, and 50.0% had received dexamethasone during their treatment, compared with none of those without fatigue (Table 1). There was no difference in serum cortisol or change in serum cortisol from baseline at any time point following Synacthen between the Fatigue and No Fatigue cohorts (Fig. 4A, Table 2). Peak cortisol at either 30 or 60 minutes following Synacthen also did not differ between the 2 groups (Fig. 4B). There was no correlation between either baseline cortisol or peak cortisol and fatigue severity or frequency (Fig. 4C-4E). Additionally, median (IQR) ACTH concentrations were not different between those who did not receive dexamethasone, compared with those who did (no dexamethasone 16.05 [9.05, 22.13] ng/L; dexamethasone 14.05 [7.53, 21.75], *P* = 0.54). No significant differences between the ACTH concentrations were seen between the Fatigue and No Fatigue cohorts: 12.35 (7.55, 21.55) ng/L and 19.15 (14.03, 22.80) respectively, *P =* 0.06.

### Thyroid Function After COVID-19

**Figure 5.**
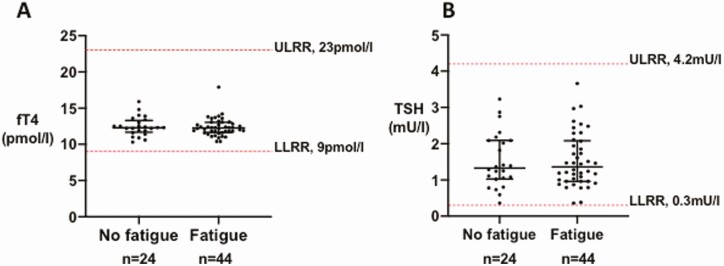
Thyroid function tests in patients with persistent fatigue. (A) Graph of individual TSH (mU/L) values in patients with fatigue (n = 44) compared with those without (n = 24), in those with no preexisting thyroid disease (n = 68). Individual data points plotted with horizontal line representing median, whiskers represent upper and lower interquartile range (IQR). The normal reference range for TSH using Abbott Architect assay (0.3-4.2 mU/L) is depicted using the red dashed line. Abbreviations: LLRR, lower limit reference range; ULRR, upper limit reference range. (B) Graph of individual fT4 (pmol/L) values in patients with fatigue (n = 44) compared with those without (n = 24) in those with no preexisting thyroid disease (n = 68). Individual data points plotted with horizontal line representing median, whiskers represent upper and lower IQR. The normal reference range for fT4 using Abbott Architect assay (9-23 pmol/L) is depicted using the red dashed line. Abbreviations: LLRR, lower limit reference range; ULRR, upper limit reference range.

In patients without preexisting thyroid disease (n = 68), levels of TSH, fT4, and fT3 were within the reference range for all patients attending ≥ 3 months after presentation with COVID-19 (median [IQR]: TSH, 1.32 [0.97, 2.09] mU/L; fT4, 12.30 [11.65, 13.08] pmol/L; fT3 4.40 [4.08, 4.80] pmol/L; see Table 2). In the Fatigue cohort, serum TSH, fT4, and fT3 did not differ from the No fatigue cohort (Table 2, Fig. 5A and 5B).

## Discussion

To our knowledge, this is the first detailed assessment of adrenal and thyroid function in patients ≥3 months following COVID-19 infection. Our group recently published data demonstrating that in the acute setting, a single cortisol measurement taken within 48 hours of admission predicted mortality. We showed that there was an appropriate stress response during acute COVID-19 without any suggestion of acute adrenal insufficiency (28). However, given that adrenal insufficiency can occur following critical illness (29) we wished to determine whether patients who have survived COVID-19 have any evidence of adrenal gland dysfunction. In our study cohort, all patients had a normal response to Synacthen, achieving a peak cortisol ≥450 nmol/L, regardless of the severity of COVID-19, their antibody status, or whether they had received dexamethasone. Basal and peak cortisol levels did not differ by disease severity or by whether they experienced persistent fatigue following COVID-19. Following publication of the data from the RECOVERY trial, the use of dexamethasone became standard care for all patients admitted with COVID-19 who had an oxygen requirement. Data from our cohort suggest that administration of dexamethasone according to this protocol (ie, 6 mg once daily for a maximum of 10 days) (30) does not impair adrenal function in the medium-term, a reassuring observation given the large number of patients who will have received this regimen as part of routine treatment of COVID-19. Our data are also reassuring given that another coronavirus (SARS) has been reported to be associated with a prevalence of hypocortisolism up to 39% in a sample of a similar size (n = 61) (7). It should be noted that these authors used an older radioimmunoassay for cortisol relative to the Abbott immunoassay in this study, which has been validated against gold-standard gas chromatography–mass spectrometry (GC-MS) methods (25). Moreover, the definition of hypocortisolism used in the earlier study included either an 8 am cortisol of 138 nmol/L or a peak cortisol of 550 nmol/L after 1 µg Synacthen. We elected to use the 250-µg Synacthen test to assess hypothalamic-pituitary-adrenal (HPA) axis integrity. This is a frequently utilized test, with widely accepted, assay-specific reference ranges recommended for detection of primary adrenal insufficiency (18). While it has been suggested that the 1-µg Synacthen test may be more sensitive at detecting secondary adrenal insufficiency (31), several meta-analyses have failed to demonstrate a difference between the 2 tests (32-34). Furthermore, significant variability exists in the methodology used to draw up the 1-µg Synacthen dose, raising the possibility of inaccuracies in undertaking the test and its consequent diagnostic performance (35). The standard 250-µg Synacthen test remains the most widely used dynamic function test for assessing the integrity of the HPA axis (35, 36), and is the gold-standard test for diagnosing primary adrenal insufficiency (18, 35, 37). Given the pathophysiology of COVID-19, primary, secondary, and tertiary adrenal insufficiency were all distinct clinical possibilities, and thus the standard short synacthen test provided the most practical way to interrogate the HPA axis in its entirety. Importantly, both the 250-µg and 1-µg Synacthen tests represent supraphysiological stimuli to the adrenal glands, with both often resulting in higher cortisol responses than the physiological stressor of hypoglycemia (31), and with Mayenknecht and colleagues demonstrating 1 µg Synacthen to result in maximal stimulation of the adrenal glands (38). Thus, the 250-µg Synacthen test afforded us the most appropriate assessment of the HPA axis in its entirety, replicating standard clinical practice.

Persistent symptoms following COVID-19 are reported to affect approximately 53% to 63% of patients (15, 16) and indeed this is reflected in our cohort, where direct questioning revealed that 62.9% of patients experienced persistent fatigue following COVID-19.

Not only is disturbance of the adrenal axis frequently associated with fatigue, but dysfunction of the thyroid axis may frequently present with tiredness. Of those patients not already known to have thyroid disease (n = 68), thyroid function tests (TSH, fT4, and fT3) were all within the normal range. We have also previously shown that there is a small reduction, on average, of TSH and FT4 compared to the pre-COVID-19 baseline and that there appears to be a recovery to intra-individual set-points for fT4 and TSH (39). Moreover, there was no evidence of increase in frequency of hypo- or hyperthyroidism in those patients with fatigue, compared with those without.

This study does have some limitations. During the development of this study, details of persistent symptoms including fatigue following COVID-19 were emerging (40). In the absence of any validated measure for fatigue post-COVID-19, we adopted a pragmatic approach, quantifying rates of fatigue using the “yes/no” response to the direct question of experience of fatigue, consistent with both studies investigating COVID-19 (20, 21) and other disease paradigms (22, 23). We then used an ordinal scale to quantify both the frequency of fatigue and severity of fatigue; however, such an approach, as with other measures of fatigue, is subjective. In addition, due to the single assessment of patients, we are unable to provide longitudinal data for all patients included in the study. For patients with details of their initial admission with COVID-19 illness available, we have provided data on cortisol levels taken within the first 48 hours of admission. Furthermore, given that all patients demonstrated adequate adrenal reserve and thyroid levels at over 3 months after initial illness, it is unlikely that clinically significant endocrine abnormalities would develop on more prolonged follow-up. Additionally, by excluding patients on glucocorticoid treatment, as well as other medications known to affect cortisol-binding globulin (like the combined oral contraceptive pill), it may be that some patients with adrenal and thyroid dysfunction following COVID-19 were not included in the study. However, given that administration of steroids is known to influence thyroid function tests, and interpretation of the response to Synacthen, it was necessary to exclude such patients to prevent the misclassification of patients who have survived COVID-19.

In summary, in this prospective study we assessed for the presence of adrenal and thyroid dysfunction in patients at least 3 months following COVID-19. We have shown that all patients in our cohort had adequate adrenal reserve irrespective of disease severity and dexamethasone treatment. In keeping with the literature, we have shown that a significant number of patients experience persistent fatigue after COVID-19. However, we report for the first time that these symptoms are not accounted for by overt adrenal or thyroid gland dysfunction. These findings have important implications for the clinical assessment of patients after COVID-19, an emerging field of medicine.

## Data Availability

Some or all data sets generated during and/or analyzed during the present study are not publicly available but are available from the corresponding author on reasonable request.

## Abbreviations:

ACE2: angiotensin-converting enzyme 2
ACTH: adrenocorticotropic hormone
ANOVA: analysis of variance
fT3: free triiodothyronine
fT4: free thyroxine
HPA: hypothalamic-pituitary-adrenal
IQR: interquartile range
IV: intravenous
SARS-CoV-2: severe acute respiratory syndrome coronavirus 2
TSH: thyrotropin (thyroid-stimulating hormone)
WHO: World Health Organization

## Notes

***Clinical Trial Information:*** ISRCTN registry number: ISRCTN15615697.

***Author Contributions:*** Conceptualizing of the study: S.A.C., B.K., K.M., S.C. A.N.C., A.A., T.T, and W.S.D.; collecting and recording of the demographic, clinical, and laboratory data: S.A.C., M.P, E.G.M., B.P., B.M., C.I.; analyzing the data: S.C. M.P, A.A; drafting of the manuscript: S.C., W.S.D.; and revising of all subsequent versions of the manuscript: S.C. and W.S.D. All authors read and approved the final manuscript.

## Additional Information

***Disclosures:*** The authors have nothing to disclose.
